# Short-time evaluation on intraocular scattering after implantable collamer lens implantation for correcting high myopia

**DOI:** 10.1186/s12886-020-01482-1

**Published:** 2020-06-17

**Authors:** Zhe Yu, Jun Li, Hui Song

**Affiliations:** Tianjin Eye Hospital, Tianjin Key Lab of Ophthalmology and Visual Science, Tianjin Eye Institute, Clinical College of Ophthalmology, Tianjin Medical University, Nankai University Eye Hospital, No. 4 Gansu Road, Heping District, Tianjin, People’s Republic of China

**Keywords:** High myopia, Phakic intraocular lens, Implantable collamer lens, Intraocular light scattering

## Abstract

**Background:**

To compare the intraocular scattering before and after implantation of implantable collamer lens (ICL) V4c for correction of high myopia in a short term.

**Methods:**

In this study, 38 eyes of 19 patients who underwent the implantation of ICL V4c were followed up for 3 months. Uncorrected visual acuity (UCVA), best corrected visual acuity (BCVA), objective scattering index (OSI), modulation transfer function cutoff frequency (MTF cutoff), strehl ratio (S/R), OV100%, OV20% and OV9% were measured pre- and postoperatively. Meantime, the Pseudophakic Dysphotopsia Questionnaire (PDQ) was scored to evaluate the subjective satisfaction of intraocular scattering pre- and postoperatively.

**Results:**

The UCVA were − 0.02 ± 0.06, − 0.03 ± 0.07 and − 0.04 ± 0.07 logMAR at 1 week, 1 month and 3 months postoperatively which were significantly better than those preoperatively (*P* < 0.05). The BCVA were − 0.09 ± 0.09, − 0.09 ± 0.1 and − 0.1 ± 0.11 logMAR at 1 week, 1 month and 3 months after surgery, which were better than those before surgery significantly (*t* = 15.64, *P* < 0.05). The mean OSI were 2.37 ± 1.6, 1.63 ± 0.94, 1.5 ± 0.86 and 1.43 ± 1.05 preoperatively, 1 week, 1 month and 3 months postoperatively which was found significant difference (*F* = 12.92 *P* < 0.05). No significant differences were found in MTF cut off (*F* = 0.61, *P* = 0.62), S/R (*F* = 0.58, *P* = 0.36), OV100% (*F* = 0.966, *P* = 0.65), OV20% (*F* = 0.121, *P* = 0.96) and OV9% (*F* = 1.01, *P* = 0.30) between pre- and postoperatively. The PDQ results indicated that intraocular scattering reduced at 3 months after surgery significantly (*P* < 0.05).

**Conclusions:**

The ICL V4c implantation for correcting high myopia induced less intraocular scattering and visual disorder than spectacle correction.

## Background

Today, people take more and more time to use computer and mobile phone. This situation leads to an increase in the number of patients with myopia. However, it is not comfortable to wear heavy glasses for patients. Hence, laser refractive surgery has been widely used. But corneal laser surgery is unsuitable for some patients, such as those with thin cornea. Such patients can resort to implantable collamer lens (ICL) to correct high myopia. Visian Implantable Collamer Lens ICL™ (STAAR Surgical, Nidau, Switzerland), a posterior chamber phakic intraocular lens, has been reported to be effective, predictable, and safe for hyper myopia or super high myopia correction [1, 2]. In particular, ICL v4c, with an artificial 360 μm central hole, reduces the dependence of Nd:YAG laser iridotomies or peripheral iridotomy, making surgery considerably safer and more convenient than before [3, 4]. However, some patients complain of seeing halos or glares, which are caused by scattering postoperatively. Intraocular light scattering is also an important parameter to evaluate visual quality postoperatively [5]. For this reason, except vision acuity and modulation transfer function (MTF) cut off, objective scattering index (OSI) and subjective questionnaire were evaluated in this study. The Optical Quality Analysis System™ (Visiometrics, Terrassa, Spain) was used in this study to quantitatively examine the intraocular scattering. A Pseudophakic Dysphotopsia Questionnaire (PDQ) was used to evaluate the subjective feeling of the patients.

## Methods

### Study population

A total of 38 eyes of 19 patients who underwent implantation of Visian ICL v4c (STAAR Surgical, Nidau, Switzerland) were included in this observational research. The Visian ICL v4c has an artificial 360 μm central hole. It was implanted into the posterior chamber and did not require peripheral iridotomies. The patients were chosen in accordance with the following inclusion criteria: 1. between 18 and 45 years old; 2. diagnosed with simple axial myopia; 3. refractive error remaining stable over 2 years (changed less than 0.5D in 2 years); 4. anterior chamber ≥3 mm; 5. intraocular pressure < 21 mmHg; 6. endothelial cell density > 2200 cells/mm [2]; 7. no significant history of glaucoma or other eye diseases; 8. urgent need for emmetropia without spectacles. All surgical procedures were successful, and no intraoperative or postoperative complications, such as cataract formation, pupillary block, glaucoma, or ICL removal, were observed during follow-up.

### Preoperative examinations and ICL power calculation

The ophthalmologic measurements were performed before surgery: uncorrected distance visual acuity (UCVA), best corrected distance visual acuity (BCVA), intraocular pressure (TOPCON, NCT, Tokyo, Japan), endothelial cell count (TOPCON, SP-2000P, Japan), objective scatter index (OSI), strehl ratio (S/R), modulation transfer function cut-off, and Optical Quality Analysis System (OQAS) values (OV): 100, 20, and 9% (OQAS, Visiometrics, Terrassa, Spain). The size of the ICL was dependent on the horizontal corneal diameter (white-to-white length) and the anterior chamber depth, which were measured before the surgery [6]. The horizontal white-to-white (WTW) distance and anterior chamber depth were measured by a Scheimpflug photography device (Pentacam, Oculus, Optikgerate GmbH, Wetzlar, Germany) in this study. Pentacam is a rotating Scheimpflug camera system which can take 25 cross-sectional photos through the cornea center and measure the horizontal WTW distance automatically. A quality factor was used to check the image quality and ensure accurate measurement. The horizontal WTW distance can be measured by a manual caliper or imaging devices such as Pentacam, Orbscan or IOL Master. The manual calipers and Pentacam measurement were both observed repeatable and accurate [7, 8]. IOL power was calculated with the formula provided by the manufacturer and aimed to emmetropia in all eyes.

### Surgical procedures

All surgeries were performed by the same experienced surgeon (Dr. H.S.). Pupils were sufficiently dilated with tropicamide (Santen Pharmaceutical Co., Ltd., Osaka, Japan) before surgery. After topical anesthesia was achieved with proparacaine (Ruinian Best Pharmaceutical Co., Ltd., Nanjing), the foldable ICL was inserted into the posterior chamber through a 3.0 mm corneal incision with a particular design injector. The ICL was placed in the ciliary sulcus and rolled to a suitable angle by using a gauge. Afterward, the remaining viscoelastic agent in the anterior chamber was removed in case of postoperative ocular hypertension. Steroidal (0.1% fluorometholone, Santen, Osaka, Japan), antibiotic (0.3% levofloxacin, Santen, Osaka, Japan), NSAIDs (Pranoprofen, Senju Pharmaceutical, Japan), and sodium hyaluronate eye drops (Santen, Osaka, Japan) were administered and reduced gradually for a month.

### Follow-up measurement and questionnaire

UCVA, BCVA, IOP, endothelial cell count, OSI, S/R, and MTF cut-off were measured at 1 week, 1 month, and 3 months after surgery. The residual spherical or cylindrical errors were corrected by using the external lens for an accurate record. Pseudophakic Dysphotopsia Questionnaire was used to evaluate the subjective satisfaction during the follow-up appointments. In this survey, the patients were asked to rate their satisfaction from 0 to 10, representing no effect to severe effect [9]. The nine questions included in the questionnaire mainly considered the evaluation of scattered light, halo, glare, and visual quality of the patient’s daily life after ICL implantation.

### Statistical analysis

The data were expressed as mean ± SD, The Visual acuity was recorded as the logarithm of the minimum angle of resolution. All the data were analyzed with SPSS Statistics 19.0 (SPSS Inc., Chicago, US) and tested with a Kolmogorov–Smirnov test. Repeated measures were performed to compare the difference between preoperative and postoperative data. The relation between the two sets of data was examined via Spearman’s correlation test. *P* < 0.05 was considered statistically significant.

## Result

The patients’ demographic data is shown in Table 1. Preoperatively, the mean age of patients was 26.11 ± 6.38 (range of 19 years to 39 years), mean spherical refraction was − 10.62 ± 3.18 (range of − 22.00 to − 5.25) D, mean cylinder was 2.17 ± 28.87 (range − 3.5 to 4.5) D, and the mean axial length was 27.50 ± 1.53 (range of 25.50 to 32.25)mm (Table 1).

**Table 1:** Patient demographic and characteristics

Age	26.11 ± 6.38
Spherical refraction (D)	−10.62 ± 3.18
Cylinder (D)	2.17 ± 28.87
AXL (mm)	27.50 ± 1.53

*AXL* axial length.

All surgical procedure was performed uneventfully. No obvious postoperative complications like pupil block or cataract formation occurred and no one lost during the 3-month follow-up. We found the spherical equivalent decreased from −10.77 ± 3.00D (range − 21.5 ~ − 5.5D) to − 0.856 ± 0.6 (range − 1.00 ~ 1.25) (*P* < 0.05). The mean pre- and postoperative UCVA at 3 months were 1.62 ± 0.247 logMAR (range 1.0 ~ 2.0) and − 0.04 ± 0.08 logMAR (range − 0.18 ~ 0.1) (t = 40.50 *P* < 0.05), respectively. And the mean pre- and postoperative BCVA at 3 months was 0.02 ± 0.05 logMAR (range, 0 ~ 0.15), − 0.20 ± 0.07 logMAR (range − 0.3 ~ 0) (t = 15.64 *P* < 0.05). The nature pupil size tested by OQAS system increased from 5.28 ± 1.23 mm before surgery to 5.69 ± 1.10 mm, 5.87 ± 0.93 mm and 5.96 ± 0.92 mm at 1 week, 1 month and 3 months after surgery under significant statistically difference (*F* = 5.82 *P* = 0.02). There was no significant difference in the IOP between pre- and 3 months postoperatively (Table 2).

**Table 2:** Demographic data of patients before and after surgery

Age (years)	26.11 ± 6.38 (19 ~ 39)			
Gender (%female)	84			
	Preoperative	Postoperative	*t*	*P*
SE (D)	−10.77 ± 3.00	−0.86 ± 0.60	−23.35	< 0.05*
UCVA (LogMAR)	1.62 ± 0.24	−0.04 ± 0.08	40.5	< 0.05*
BCVA (LogMAR)	0.02 ± 0.05	−0.2 ± 0.07	15.64	< 0.05*
IOP (mmHg)	14.53 ± 3.64	15.79 ± 3.86	−1.52	> 0.05
Pupil size (mm)	5.28 ± 1.23	5.96 ± 0.92	−3.16	< 0.05*

*SE* Spherical Equivalent, *D* diopter, *UCVA* uncorrected visual acuity, *BCVA* best corrected visual acuity, *IOP* intraocular pressure P* means significant difference.

The mean vaults were 469.08 ± 125.09 μm, 456.84 ± 123.94 μm and 449.87 ± 132.08 μm at 1 week, 1 month and 3 months postoperatively which was found no significant difference among them (*F* = 2.749 *P* > 0.05). There was also no significant relationship between vault and nature pupil size at each time node (*r* = 0.03, − 0.01,0.15 *P* > 0.05).

The safety index (mean postoperative BCVA/mean preoperative BCVA) was1.30, 1.32, and 1.37 at 1w, 1 m and 3 m after surgery and no eye lost 1 or more line. Six eyes (15.7%) didn’t change in the BCVA, 18 eyes (47.5%) gained 1 line and 14 eyes (36.8%) gained more than 1 line postoperatively.

The efficacy index (mean postoperative UCVA/mean preoperative BCVA) was 1.09,1.14, 1.15 at 1w, 1 m and 3 m, postoperatively. The spherical equivalent changed significantly from − 10.77 ± 3.00 to 0.86 ± 0.60 3 months postoperatively (t = − 23.35 *P* < 0.05). The mean BCVA changed from 0.02 ± 0.05 logMAR to − 0.2 ± 0.07 logMAR significantly (t = 15.64 *P* < 0.05).

**Fig. 1:**
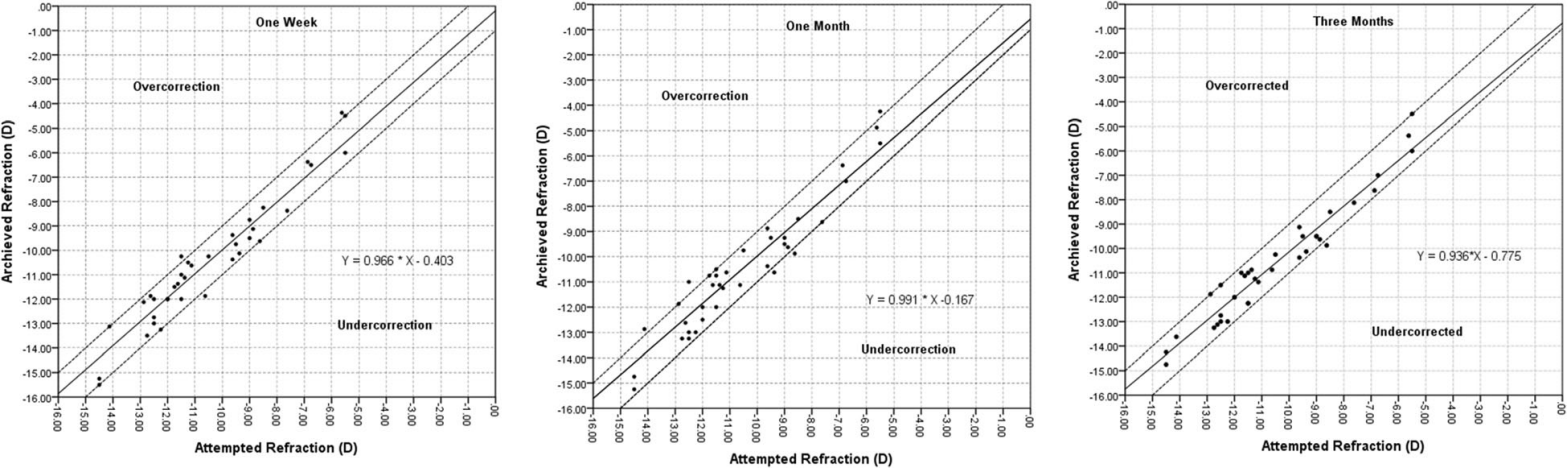
Postoperative predictability of ICL implantation at 1w, 1 m and 3 m postoperatively

Predictability: At 3 months after surgery, 13 eyes (34.2%) refractive error were within ±0.25D, 33 eyes within ±0.75D (86.8%) and 37 eyes within ±1D (97.4%) from attempted refraction. R^2^ was 0.946,0.931 and 0.961 at 1w, 1 m and 3 m. (Fig. 1).

**Fig. 2:**
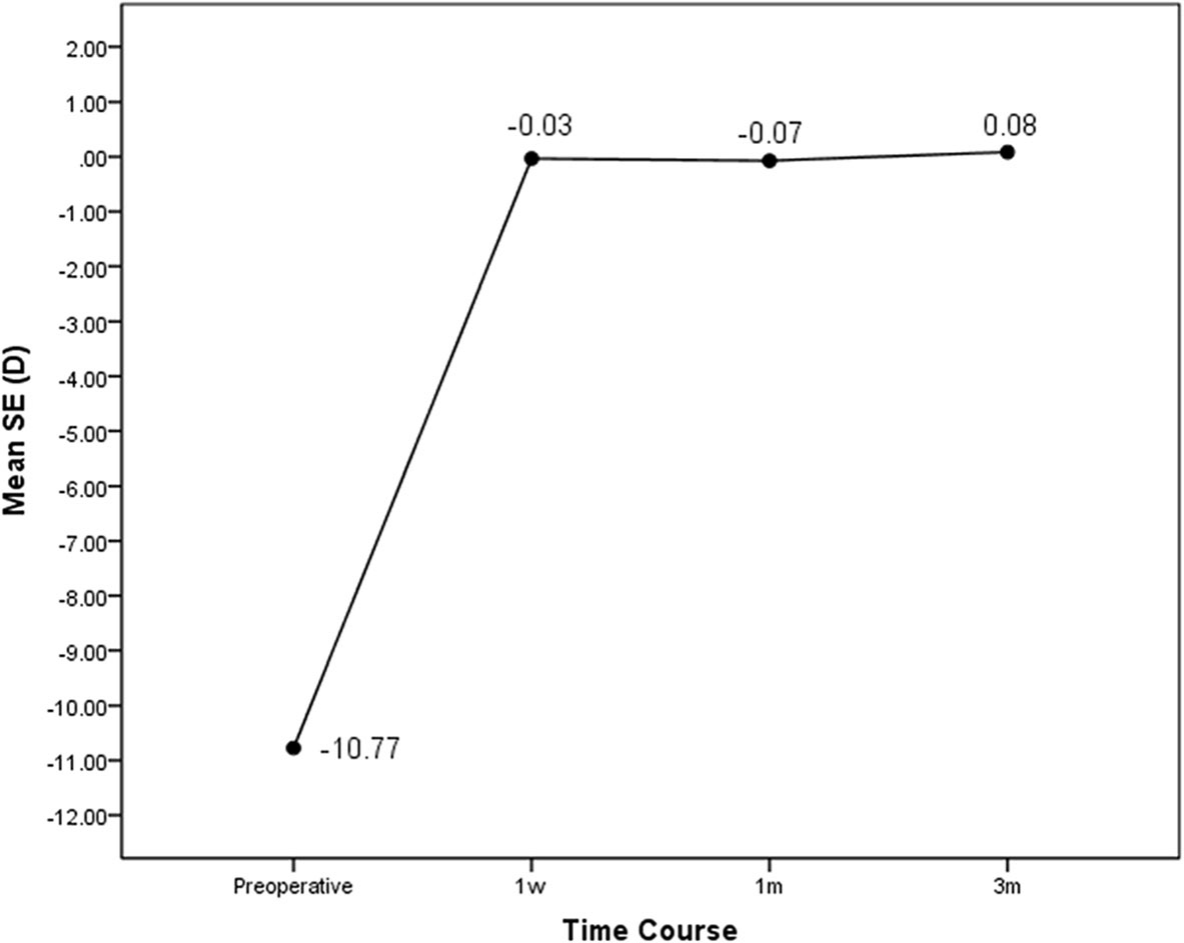
Stability of ICL implantation at pre operatively, 1w, 1 m and 3 m postoperatively

Stability: The change in mean SE from 1 week to 3 months was not statistically significant (F = 1.99 *p* = 0.15) with − 0.03 ± 0.7, − 0.07 ± 0.78, 0.08 ± 0.6 at 1w, 1 m and 3 m after ICL implantation, respectively (Fig. 2).

The mean OSI values before and 1 week, 1 month, 3 months after surgery were 2.37 ± 1.6 (range 0.3 ~ 7.5), 1.63 ± 0.94 (range 0.5 ~ 4.0), 1.5 ± 0.86 (range 0.5 ~ 3.9) and 1.43 ± 1.05 (range 0.3 ~ 5.7), respectively. A significant decrease was founded (*F* = 12.92 *P* < 0.05). However, there was no difference among three follow-ups after the surgery (*P* = 0.24, 0.7, 0.56). We found no significant difference in the MTF cutoff frequency (*F* = 0.61, *P* = 0.62) or the strehl ratio (*F* = 0.58, *P* = 0.36) from the beginning to the end, respectively. No significant difference was found in OV100%, OV20% and OV9% between before and after surgery (*F* = 0.97, *P* = 0.65), (*F* = 0.12, *P* = 0.96), (*F* = 1.01, *P* = 0.30) (Table 3).

**Table 3:** The parameters of OQAS test before and after ICL implantation

	Preoperation	1 week	1 month	3 months	*F*	*P*
OSI	2.37 ± 1.6	1.63 ± 0.94	1.5 ± 0.86	1.43 ± 1.05	12.92	P^*^ = 0.00
S/R	0.16 ± 0.07	0.16 ± 0.04	0.16 ± 0.04	0.17 ± 0.05	0.582	*P* ***=*** 0.60
MTF	28.74 ± 12.77	29.33 ± 8.7	29.72 ± 8.49	31.19 ± 9.52	0.609	*P* = 0.58
OV100%	0.07 ± 0.22	0.06 ± 0.28	0.08 ± 0.24	0.13 ± 0.34	0.966	*P =* 0.39
OV20%	0.23 ± 0.23	0.21 ± 0.18	0.22 ± 0.16	0.22 ± 0.21	0.121	*P =* 0.93
OV9%	0.45 ± 0.25	0.42 ± 0.12	0.42 ± 0.15	0.39 ± 0.16	1.013	*P =* 0.37

*OSI* objective scatter index, *S/R* strehl ratio, *MTF* Modulation Transfer Function cutoff frequency, *OV* OQAS value, P* means significant difference.

Meanwhile, assessment of subjective intraocular light scattering after surgery was based on the PDQ scale (Table 4). All nine questions had a significant difference among the 1 week, 1 month and 3 months after surgery (*P* < 0.05). We found the significant difference in the questions about “bright in general, flashes avoid light and side/above light” between 1w and 1 m postoperatively (*P* < 0.05). A significant difference was found in the question “the bright light in general and headlights at night” between 1 m and 3 m after surgery (*P* < 0.05). All the nine answers had significant difference between 1w and 3 m after surgery (*P* < 0.05). Compared with the OSI, the PDQ mean scale had no significant relationship whatever at 1w (*P* = 0.06), 1 m (*P* = 0.19) and 3 m (*P* = 0.43) after surgery (Table 5).

**Table 4:** The rate of PDQ questionnaire

PDQ question	1 week	1 month	3 months	*F*	*P*^*0*^
1 bright light in general	6.11 ± 1.94	3.63 ± 2.18	1.63 ± 1.38	25.941	=0.00*
	***P***^**1**^ = 0.00*	***P***^**2**^ = 0.01*	***P***^**3**^ = 0.00*		
2 headlights at night	5.53 ± 1.57	4.21 ± 1.94	2.11 ± 2.2	14.571	=0.00*
	*P*^*1*^ = 0.13	***P***^***2***^ = 0.01*	***P***^***3***^ = 0.00*		
3 halos around light	5.53 ± 2.39	4.05 ± 2.39	2.21 ± 2.48	8.457	=0.00*
	*P*^*1*^ = 0.22	*P*^*2*^ = 0.08	***P***^***3***^ = 0.00*		
4 flashes avoid light	4.26 ± 2.55	2.47 ± 1.79	0.95 ± 1.19	13.370	=0.00*
	***P***^***1***^ = 0.02*	*P*^*2*^ = 0.63	***P***^***3***^ = 0.00*		
5 dark/grey shadow	2.84 ± 3.22	1.53 ± 2.21	0.79 ± 1.54	3.317	=0.04*
	*P*^*1*^ = 0.33	*P*^*2*^ = 1.00	***P***^***3***^ = 0.04*		
6 glares blind me	2.58 ± 2.56	1.21 ± 1.67	0.58 ± 0.88	5.573	=0.01*
	*P*^*1*^ = 0.09	*P*^*2*^ = 0.92	***P***^***3***^ = 0.01*		
7 side/above light	3.16 ± 2.21	1.11 ± 1.29	0.89 ± 1.55	9.429	=0.00*
	***P***^***1***^ = 0.00*	*P*^*2*^ = 1.00	***P***^***3***^ = 0.00*		
8 flickering shadow	2.84 ± 3.01	1.37 ± 2.23	0.68 ± 1.42	4.090	=0.02*
	*P*^*1*^ = 0.18	*P*^*2*^ = 1.00	***P***^***3***^ = 0.02*		
9 semi-circular shadow	1.79 ± 2.28	0.68 ± 1.03	0.21 ± 0.52	5.414	=0.01*
	*P*^*1*^ = 0.09	*P*^*2*^ = 1.00	***P***^***3***^ = 0.01*		

The minimum score for each question was 0 (no symptoms) and the maximum score was 10 (feel the worst with symptoms) *P*^*0*^ = difference among 1w, 1 m and 3 ml; *P*^*1*^ = difference between 1w and 1 m; *P*^*2*^ = difference between 1 m and 3 m, *P*^*3*^ = difference between 1w and 3 m; P* means significantly difference.

**Table 5:** The relationship between the PDQ scale and 4 mm OSI tested by OQAS postoperatively

	1 week	1 month	3 months
Score	3.84 ± 2.86	2.25 ± 2.3	1.12 ± 1.7
OSI	1.63 ± 0.94	1.5 ± 0.85	1.43 ± 1.05
*r*	0.3	0.2	0.13
*P*	0.06	0.19	0.43

Score = PDQ mean score.

## Discussion

According to our study, the patient’s postoperative spherical equivalent was significantly reduced and remained stable 3 months after ICL implantation. UCVA and BCVA were significantly improved, and a stable intraocular pressure level was maintained until the 3rd month without iridotomy [10]. The efficacy, safety, stability, and predictability of the ICL v4c type in clinical applications were satisfactory, and this finding was similar to previous results [11].

Our main research goal was to compare the effects of spectacle correction and ICL correction in patients’ objective and subjective intraocular scattering. The effect of artificial central hole in intraocular scattering was also considered in investigation. The postoperative visional quality, high-order aberrations, and contrast sensitivity were assessed in some studies [12–14]. In this study, the intraocular scattering value was examined via OQAS and the PDQ scale as important aspects that affected the objective and subjective feelings of the patients postoperatively. The OSI detected by OQAS is the ratio of the peripheral energy to the central energy of the image recorded by the retina reflecting from the instrument. According to our research, the significant difference between the OSI values under the preoperative and postoperative conditions indicated that the ICL implantation could reduce intraocular scattering more properly compared with spectacle correction for high myopia. This also indicated that the central hole might not influence the scatter value of eyes [15]. Shiratani and Uozato et al. [16, 17] also found that the 360 μm hole is the most suitable size for aqueous flow, which does not affect intraocular scattering. Spectacles are unstable and always exposed to air, resulting in the deviation of the viewing axis and the lens axis. However, the ICL is implanted in the eye and fixed in the ciliary sulcus through the accurate selection of the lens’ size by accurately measuring the white-to-white length before surgery, ensuring the transparency of the refractive media, minimal decentration, and tilt to reduce intraocular scattering [18]. Other study suggested that the distance between the lens and the retina can also cause scatter difference [19].

Our PDQ results indicated that the patients were found to experience visual changes, such as the presence of glare and halos, in the early postoperative period. Over time, such as 3 months, they felt that the relevant visual effects caused by intraocular lens implantation reduced and became stable gradually. These visual defects, such as glare or halo, may be caused by the changes in the eye-use pattern of a patient in the early postoperative period [20]. We thought such effects are reduced by adaptation. The gradual improvement of subjective feeling also confirmed that the artificial central hole does not have an obvious effect on the intraocular scattering [21]. However, six eyes (15.8%) felt that visual disorder still troubles them at night. Dick, Franssen et al. reported that the pupil diameter affects the occurrence of halos and glare, which is not correlated with straylight values [22, 23]. As we concern that the OSI measured by OQAS was under the 4 mm artificial pupil mode. The mean nature pupil size is more than 5 mm in dark circumstance, which possibly caused a deviation in OSI and PDQ scales [24, 25].

The diameter of the natural pupil increases postoperatively, which is inconsistent with previous results [26, 27]. A slightly larger crystal diameter was selected preoperatively to ensure that the ICL was fixed in the ciliary sulcus. This slightly larger ICL diameter led to a slight bending of the lens and increase in pupil size. However, according to our analysis of relationship between pupil size and vault, there was no direct evidence supported this observation; thus, this aspect should be studied further.

No significant difference was observed in MTF cut off and S/R between spectacles and ICL group in our study. The pre- and postoperative OV 100, 20, and 9% (Simulated Contrast Vision) did not change significantly. S/R reflected the ratio between the point spread function peak value with and without the optical aberration system [28]. Our result indicated that the ICL implantation did not induce extra optical aberration to the whole refractive system. Hence, MTF and S/R remained stable, indicating that no significant change occurred if the fundus condition remained constant. Kamiya and Qin’s study indicated that these data were consistent for a certain period after ICL implantation, and their finding was similar to our results [29, 30].

The transverse diameter of ICL size is determined base on the anterior chamber depth and horizontal WTW. The measurement errors of the two parameters lead to abnormal vault and ICL misalignment (such as decentration and tilt). Seo and associates found WTW correlated closely to the postoperative ICL vault [31]. Excessive high vault leads to angle block [32]. Low vault can result in anterior subcapsular cataract [33].

And the ICL misalignment is closely correlated with abnormal vault [34]. Cari and associates found the ICL decentration could significantly increase the coma, but had little effect on point spread function. But the ICL decentration was only 0.6 mm in Cari’s study [35]. Previous studies have shown the IOL decentration and tilt can markedly affect the visual quality including MTF and ocular aberration. Liu and associates found the misalignment of a multifocal IOL markedly decreased the visual quality [36]. Pérez-Gracia and associates found the misalignment of aspheric IOLs could increase the ocular aberrations and affect the MTF [37]. Taketani and associates found there was a stong correlation between coma and IOL tilt [38]. So the inaccurate measurement of WTW may affect the visual quality after ICL implantation. And further study is needed.

The major limitation of this study is the limited sample and insufficient follow-up time. In our previous opinion, the intraocular scattering becomes stable gradually at 1 month after IOL implantation [39]. Moreover, the artificial pupil was limited to 4 mm during the OQAS test; thus, the diameter of natural pupil was not simulated. Therefore, obtaining a relatively comprehensive relationship between pupil and intraocular scattering values is difficult.

## Conclusions

The ICL with central hole is a safe and effective surgery for patients for high myopia correction. The intraocular scattering induced by ICL is less than spectacles and unaffected by the presence of the central hole.

## Data Availability

The data of this study are available from the corresponding author.

## Abbreviations

ICL: Implantable Collamer Lens
OQAS: Optical Quality Analysis System
UCVA: Uncorrected visual acuity
BCVA: Best corrected visual acuity
SE: Spherical Equivalent
D: Diopters
IOP: Intraocular pressure
OSI: Objective scattering index
MTF cutoff: Modulation transfer function cutoff frequency
S/R: Strehl ratio
NSAIDs: Nonsteroidal Anti-inflammatory Drugs
PDQ: Pseudophakic Dysphotopsia Questionnaire
Nd:YAG: Neodymium - yttrium - aluminum – garnet
SD: Standard deviation
SPSS: Statistical Product and Service Solutions
